# Supplementary material for: Learning curve and surgical outcome of robotic assisted colorectal surgery with ERAS program
Source: Sci Rep. 2022 Nov 29;12:20566. doi: 10.1038/s41598-022-24665-w (PMC9709162; doi:10.1038/s41598-022-24665-w)
Supplement: Supplementary file 2 — Supplementary Information 2. [file 41598_2022_24665_MOESM2_ESM.zip › RAL Raw data-Ñ[▒K/135/Patho_ñw╝╨░O▒Kñσ.pdf]

臺中榮民總醫院 臺中市臺灣大道四段1650號 病理部活體組織切片 臨床醫學實驗及病理診斷中心 檢驗(查)報告

[redacted]

Pathologic diagnosis:
1. Sigmoid colon, robot assisted colectomy --- a. Chronic colitis with submucosal edema. No tumor is found. b. Unremarkable mesocolic lymph nodes (0/17).
2. IMA root, dissection --- Unremarkable mesocolic lymph node (0/1).
3. Colon, cut end, distal, resection --- Unremarkable.

Ancillary study for diagnosis:
Recut done for section G and H.

Gross description:
The specimen consists of 1) a segment of colon, 10 cm in length and 5 cm in proximal cut end circumference, 7 cm in distal circumference, and mesocolic soft tissue. There is no tumor seen grossly. A stitch labeled the previous polypectomy site is seen, 3 cm away from the distal cut end. There are also 2 tiny mucosal buldging, up to 0.3x0.3 cm, noted 3 cm to the distal cut end. The both cut ends are free of tumor grossly. 2) IMA root, a piece of tan yellow soft tissue, 5x3.5x2 cm. 3) distal cut end, a piece of tan mucosa-covered soft tissue, 2.5x2.5x1 cm. Representative part for section: A) spcimen 2 B) specimen 3 C) proximal cut end D) distal cut end E) radial margin F) tiny mucosal buldging G-J) stitch site (inked by yellow dye) K-L) lymph nodes.
#T-59470_2 #M-43000_2 0 1105 000000
#T-C4560_2 #M-09183_2 0 1105 000000

已電子簽章

敬告：本單僅供醫師參考使用，不具任何申請效力！！
請病患妥為保管，若不慎遺失需自負個人資料外洩責任！
